# Nurturing Sustainability in Toddlerhood: Investigating Preschool Teachers’ Views and Daily Practices in a Swedish Preschool

**DOI:** 10.3390/children11121412

**Published:** 2024-11-22

**Authors:** Deniz Kahriman-Pamuk, Ingrid Pramling Samuelsson

**Affiliations:** 1 Department of Early Childhood Education, Faculty of Education, Mersin University, Mersin 33160, Türkiye; 2 Department of Education, Communication and Learning, University of Gothenburg, 405 30 Gothenburg, Sweden; ingrid.pramling@ped.gu.se

**Keywords:** early-childhood education for sustainability (ECEfS), toddlers, daily practices, case study

## Abstract

Background/Objectives: While studies suggest that young children can learn and think about sustainability, most research has focused on children aged three to five, leaving a significant gap in understanding how to engage toddlers (one to three years of age) in early-childhood education in sustainability (ECEfS). This study aims to address this gap, a crucial and often overlooked area, by investigating preschool teachers’ views of ECEfS and their daily practices for nurturing sustainability in a toddler group in a Swedish preschool. Methods: We employed a case study approach, incorporating observations, interviews, and document analysis for data collection. Qualitative techniques were used for data analysis. The research was conducted with a strong commitment to ethical considerations, including obtaining participants’ consent, ensuring confidentiality and privacy, informing them of their right to withdraw, and maintaining anonymity. Results: The findings demonstrate that the teachers possessed a solid understanding of education for sustainability and were capable of effectively implementing sustainability-related daily practices in toddler groups. By creating suitable learning environments and integrating sustainability issues into daily classroom activities, they enhanced the children’s overall learning experiences. Conclusions: This study’s findings underscore the pivotal role of educators in shaping the mindsets and behaviors of future generations, thereby supporting the long-term goals of sustainable development. By nurturing toddlers’ innate curiosity and harnessing their brains’ plasticity, educators can effectively engage toddlers in ECEfS and promote sustainable development from an early age. This study’s implications highlight the need to prioritize ECEfS during toddlerhood to realize the full potential of this critical investment in the future of our planet.

## 1. Introduction

The climate crisis, mass biodiversity loss, pollution, pandemics, extreme poverty, inequality, violent conflicts, and other environmental, social, and economic crises threaten life on Earth [1]. These interconnected challenges spur an urgent call for immediate and comprehensive action, as they jeopardise the existence of life on this planet. Governments, global and local organisations, civil society, younger generations, academia, and educational institutions agree that immediate action is needed to address these challenges [2]. While this perspective underscores the interconnectedness of these challenges, some scholars argue that treating these issues as being inherently interconnected may oversimplify their unique complexities. For instance, Easterly (2006) contends that addressing each challenge individually allows for more targeted and effective interventions, as the factors contributing to each issue can be vastly different [3]. Similarly, Collier (2007) argues that blanket solutions to global problems often fail because they do not account for the specific social, economic, and cultural contexts of individual issues [4]. Acknowledging this debate enriches the discourse on global challenges and highlights the importance of tailored strategies in addressing them.

Education for sustainability (EfS) is recognised as one of the most powerful tools for addressing these complex challenges. EfS is an emerging, developing, and dynamic phenomenon that is arising in response to the world’s needs, inviting people of all ages to take responsibility in their societies and become pioneers for sustainable futures [5]. It is critical in shaping individuals’ knowledge, values, and behaviours. It ultimately promotes sustainable practices integrating economic, social, and environmental dimensions to secure a sustainable future for all species. EfS should encompass holistic, collaborative, participatory, and interdisciplinary approaches and begin with early-childhood education [6,7]. Davis and Elliott (2023) describe EfS as positive, future-oriented, learner-centred, community-connected, lifelong, interdisciplinary, and transformative [8]. Early childhood, defined as the period from birth to eight years old, is widely recognised as a critical developmental phase for internalising values, habits, and behaviours related to sustainability and shaping future actions [9].

Early-childhood education, in particular, is seen as a “natural starting point” for lifelong learning in sustainability due to its foundational role in developing a sustainable mindset. In this context, early-childhood education for sustainability (ECEfS) is vital for empowering children to contribute to a sustainable society, as the attitudes, values, knowledge, and experiences formed in early childhood can have lasting impacts [10]. Recent research has shown that children can express opinions on environmental and social problems even at very young ages [11,12]. However, according to Piaget’s theory of cognitive development, toddlers are in the preoperational stage (ages 2 to 7), characterized by limitations in logical reasoning and understanding abstract ideas [13]. This indicates that while young children can express concerns, they may struggle to grasp complex concepts like sustainability fully. In light of this, the most important point in EfS practices during the preschool period is the concretisation of abstract concepts by integrating them with the existing education program [14]. By doing so, educators can make sustainability concepts more accessible to young learners. Additionally, by acknowledging children’s agency—that is, their capacity to act independently and make their own choices—educators can design learning experiences that translate abstract sustainability concepts into concrete, tangible activities. This approach not only aligns with their developmental needs but also empowers young children to be active participants in sustainability practices [15]. While research has confirmed that children can engage meaningfully with sustainability concepts [11], much of this research has focused on preschoolers aged three to five. This focus has created a notable gap in understanding how to introduce sustainability to toddlers aged one to three. Limited research exists on how ECEfS is applied during toddlerhood, suggesting that more attention needs to be paid to how toddlers can also benefit from education for sustainability [16].

Toddlerhood represents a pivotal time for establishing habits and values that can influence a child’s future. Toddlers, typically counted as children one to three years of age, are at a critical stage of cognitive and intellectual growth [17]. By nurturing toddlers’ natural curiosity and leveraging the brain’s plasticity during this developmental phase, educators can shape their behaviours and mindsets to align with the long-term goals of sustainability. Toddlers learn concepts through interactions with their environment, forming intuitive understandings of daily experiences [18]. This developmental window provides an opportunity to foster sustainability through everyday activities and routines, a critical investment in their future roles. For instance, routines such as diaper changing, dressing, and mealtimes can be transformed into meaningful learning opportunities for toddlers [19].

Outdoor play is another critical medium for introducing sustainability concepts to toddlers. Allowing toddlers to play outdoors provides them with opportunities to engage with nature. Observing them make mud potions, collect twigs and rocks, or observe ants carrying leaves can serve as an introduction to education for sustainability [20]. Through these playful interactions with nature, toddlers develop a relationship with their environment, which is foundational for future environmental responsibility [21].

For early-childhood educators, teaching toddlers about sustainability in age-appropriate ways means building on everyday moments found in outdoor play [20,22]. While toddlers may not fully understand the broader scientific implications of sustainability, their sensory experiences—touch, smell, sound, sight, and taste—form the basis for early environmental learning. These experiences can be harnessed to teach young children the importance of caring for the environment, laying the foundation for a deeper understanding and appreciation of the natural world. This, in turn, fosters a generation that values and prioritises sustainability [20].

Introducing sustainability practices during this formative period is essential for establishing lifelong values and behaviours [10]. These practices become normalised by integrating sustainability into toddlers’ daily routines, such as water conservation or reducing waste [23]. When sustainability is ingrained in toddlers’ daily lives, they are likelier to maintain these habits into adulthood. This early exposure can shape their mindsets, and encouraging these actions to contribute to a sustainable future [16]. Activities like nature walks, gardening, and upcycling further cultivate an appreciation for the environment and emphasise the importance of its preservation [12]. Thus, the role of the teacher becomes crucial in ensuring that these moments of play and routine foster a deep-rooted connection to sustainability.

Understanding these perspectives is crucial for guiding this work as a contribution of new evidence supporting ECEfS. By acknowledging the challenges and debates surrounding the interconnectedness of global issues and the introduction of complex concepts to toddlers, this study aims to explore practical methods for integrating sustainability into toddler education. It leverages their natural curiosity and everyday experiences to foster a foundational understanding and appreciation for the environment, thereby contributing valuable insights to the field.

### Teachers Working with Toddlers

The role of teachers in early-childhood education, particularly in fostering learning for toddlers, is central. While toddlers are naturally capable learners, they benefit greatly from the guidance and support of teachers [24]. This support extends to the development of sustainability skills and competencies. Teachers are the architects of early sustainability learning, modelling behaviours, and embedding sustainable practices in daily routines. Preschool teachers play a pivotal role in ECEfS, acting as facilitators and leaders who influence young children’s attitudes, values, knowledge, and experiences [25]. As role models, their attitudes toward sustainability directly impact the children they teach. Teachers’ perspectives on sustainability and their daily practices are critical, as there is a direct link between their approaches and promoting sustainability in education [26,27].

Research has demonstrated that preschool teachers’ understanding of and views on sustainability significantly impact the activities they implement in the classroom [28,29]. This highlights the need to further explore the connection between teachers’ perspectives and their sustainability-related practices, particularly when working with toddlers [30]. However, scholars note a research gap concerning toddlers that has led to a lack of understanding of how education for sustainability can be applied in these early years [16,31]. Exploring how teachers approach sustainability with respect to toddlers can yield new insights that contribute to practice and policy, filling this crucial gap in the literature.

Therefore, this study addresses this gap by investigating preschool teachers’ perspectives on sustainability and their sustainability-fostering practices applied to toddlers in a Swedish preschool. By investigating this topic, this study aims to contribute to our understanding of how to effectively engage toddlers in ECEfS, promoting sustainable development from an early age, which is vital for building a more sustainable future. Thus, prioritising ECEfS during toddlerhood is crucial to fully realise the potential of this critical investment in our planet’s future. In this regard, the specific research questions are as follows:

1. What are the views of preschool teachers on ECEfS in relation to a toddler group at the Swedish preschool?
2. What are the daily practices used to foster sustainability concepts in toddlers at the Swedish preschool?

## 2. Materials and Methods

### 2.1. Ethical Considerations

The researchers upheld a balanced subjective–objective perspective to ensure a comprehensive, transparent, and objective analysis. This study was conducted meticulously, with results reported accurately and without manipulation [32]. All participants received detailed information about this study’s aims, methods, and potential implications, ensuring they could make informed decisions about their involvement. All participating teachers were fully informed about the research process and willingly consented to participate. In the case of child participants (toddlers), written consent was obtained from their parents or legal guardians, guaranteeing their rights and welfare were prioritized throughout the research process. During observations, the researchers approached both children and teachers with consideration and respect. To maintain anonymity, each interview was assigned a unique number, and all data were securely stored in a locked folder on the researchers’ computers. Confidentiality measures were strictly enforced, with identifying details removed or coded to protect the privacy of participants and the preschool setting. Participants were also informed of their right to withdraw from the study at any time without any negative consequences. By ensuring these measures, the researchers remained committed to maintaining the highest ethical standards, safeguarding the well-being and rights of all participants involved in the study.

### 2.2. Research Setting and Participants

Sweden’s Early-Childhood education and Care (ECEC) system follows the Nordic model, offering a unified approach that does not differentiate between childcare and preschool [31]. The Swedish national curriculum emphasises education for sustainability for children aged 1–6 [33].

The study was conducted at a purposefully selected preschool in a large city in Sweden. This preschool was chosen due to its strong emphasis on ECEfS and its unique location adjacent to a large natural area, which provides children with easy access to the forest and supports outdoor learning and nature-based activities. Established in 2016, the preschool operates in a long, single-story building with six departments arranged in a row. Each department has its unique focus and activities. Among several potential schools, this preschool stood out for its exemplary integration of sustainability into its curriculum and daily practices, making it particularly suitable for our research objectives. This research focused specifically on the toddler group, which comprised 15 children.

To select participants for this study, purposeful criterion sampling was employed. The inclusion criteria specified that participants had to be teachers who worked with a toddler group and prioritized sustainability in their teaching practices. Three teachers who demonstrated a strong commitment to sustainability, aligning with the goals of this study, met the criteria and participated in the study. Please see Table 1 for more details about the participants.

### 2.3. Data Collection Techniques

This study employed various data collection methods, including interviews, observations, and document analysis, to gather comprehensive insights about preschool teachers’ views and daily practices related to EfS. The data collection process began with interviews, followed by concurrent observations and document analysis, allowing for triangulation of data and greater depth of understanding.

#### 2.3.1. Interviews

A semi-structured interview guide was developed to explore the teachers’ views and daily practices related to EfS. Two experts specializing in qualitative research and EfS reviewed the preliminary interview questions to ensure content validity. After incorporating their feedback, a pilot interview was conducted to refine the questions, resulting in nine open-ended questions for the main interviews [34]. An example question from the final set is “Could you please explain the difficulties you face while performing daily ECEfS practices with a toddler group?”.

Face-to-face interviews with the three preschool teachers were conducted in November 2023, each lasting an average of approximately 40 min. All interviews were audio-recorded with participants’ informed consent, and recordings were transcribed verbatim for analysis. These interviews provided essential data on the teachers’ perspectives, challenges, and practices regarding EfS in the toddler group.

#### 2.3.2. Observations

The first author carried out unstructured observations twice a week for 20 weeks from October 2023 to February 2024 to gather data on how teachers implemented EfS in their daily routines. Each observation session lasted approximately 6 h. The researcher observed various aspects of the educational environment, including daily activities, curriculum resources, routines, and environmental setups, while taking field notes and recording reflective memos.

During these observations, the researcher acted as a non-participant observer, meaning they did not engage in the teaching process but observed and recorded without interacting with the activities [34]. This allowed for an unbiased and natural capture of the teachers’ day-to-day practices related to sustainability. The researcher approached both children and teachers with consideration and respect throughout the observations.

#### 2.3.3. Document Analysis

Document analysis supplemented and validated the data gathered from interviews and observations. This method provided additional insights, helped verify findings, and offered alternative explanations when necessary [35]. The Swedish National Curriculum for Preschool (Lpfö 18) [33] curriculum were utilised to inform the development of the interview questions, grounding the research in the national educational framework.

Various documents were analysed in the classroom, including picture books, daily schedules, and visual materials. Selection criteria for documents included relevance to sustainability practices and visibility to children. Picture books were systematically reviewed, as they were scattered across different classroom areas, including the toilet, hallway, play area, and art centre. The daily schedules were displayed prominently on the hallway walls, with some schedules and plans stored in the teachers’ documentation. Visual displays, such as reminders like “use only one paper towel after washing your hands”, were also analysed. Document analysis provided concrete examples of how sustainability practices were visually reinforced in the children’s environment.

### 2.4. Data Analysis

The data analysis process was conducted continuously throughout the data collection period and upon the study’s completion. We followed Braun and Clarke’s (2006) six-phase process for thematic analysis, which includes (1) becoming familiar with the data, (2) generating initial codes, (3) searching for themes, (4) reviewing themes, (5) defining and naming themes, and (6) producing the final report [36]. This structured approach ensured a systematic and thorough analysis of the data.

#### Data Preparation and Coding Process

Interview recordings were transcribed verbatim, and written data from observations and document analysis were compiled. The research team reviewed all the data and employed open coding, where expressions relevant to recurring patterns or themes were identified and coded. These initial codes were derived from both explicit and implicit meanings within the data.

Following the initial coding, the codes were grouped into potential themes, and data relevant to each theme were collated. The research team continually checked for internal consistency within each theme, ensuring that all codes accurately reflected the core ideas of the theme. Themes and codes were then discussed, revised, and organised, ensuring clarity and coherence in their interpretation. After organising the themes and codes, we utilized MAXQDA analytics pro 2020 (release 20.4.0), a computer-assisted qualitative data analysis software, to perform a word frequency check, identifying common terms and refining the coding process.

We aligned our coding with the OMEP ESD (Education for Sustainable Development) Rating Scale during the word frequency check, ensuring consistency with established sustainability frameworks. The official name of the rating scale is the Environmental Rating Scale for Sustainable Development in Early Childhood (ERS-SDEC). Available in nine languages (https://omepworld.org/), the ERS-SDEC was developed with inspiration from the Early Childhood Environment Rating Scale (ECERS) established by Harms, Clifford, and Cryer (1998) [37]. The OMEP ESD rating scale was developed based on best practices in early-childhood education, aimed at fostering a shared culture of sustainability among children and adults [38]. Recent research demonstrates that OMEP’s ESD Rating Scale can be employed for the professional development of preschool staff, assisting them in reflecting on, discussing, and reviewing their understandings and assessing, changing, and improving their own practices [39,40,41,42].

The Swedish-adapted version of the OMEP ESD rating scale, which has been tested and reviewed with respect to experiences by researchers and preschool teachers [42,43], was utilized for this study. It consists of three key dimensions of sustainability—economic, social, and environmental—and provides a comprehensive framework for understanding sustainable development in early-childhood settings. Using its terminology enhanced our data analysis and contributed to sustainability research and practice. The codes were assessed for their compatibility with the OMEP ESD rating scale, and categories, themes, and codes were renamed accordingly.

An external expert in ECEfS was included in the entire data analysis process to ensure rigour and credibility. Final decisions on naming themes and codes were made after discussions between researchers and the expert, considering shared and differing viewpoints on the content analysis. This collaborative process helped to ensure that the themes and codes accurately represented the data and aligned with the overarching framework of the study.

To ensure credibility, the researchers independently analysed the data, comparing and discussing their coding to reach a consensus. They revisited the data multiple times to clarify the codes [36]. Potential biases were monitored by considering alternative explanations during peer debriefing meetings. Detailed descriptions of the participants’ characteristics and research procedures were provided to ensure transferability [34,44]. Please refer to Table 2 for a summary of the data collection and analysis process.

## 3. Findings

### 3.1. Preschool Teachers’ Views

#### 3.1.1. Preschool Teachers’ Views of EfS in Toddler Groups

The preschool teachers perceived EfS primarily as preserving and nurturing nature, aligning with the environmental dimension of sustainability. Teacher 1 (T1) emphasised the urgency of integrating EfS into early education: “*We need EfS. It is very important because we have one earth and live like we have four worlds. And we only have one. So, we need to take care of it*”. T1 highlighted the importance of fostering a connection with nature and teaching children to respect all living creatures: “*Look at the lonely spider. We need to take care of it. Do not step on it*”. Similarly, Teacher 3 (T3) defined EfS as “*teaching children how to care for nature and asserting that protecting our world is achievable through simple daily actions like saving water, recycling, and picking up garbage*”. These statements reflect the teachers’ belief that instilling a sense of responsibility towards nature and sustainable practices from a young age is crucial for protecting the planet.

These statements reflect the teachers’ view that instilling a sense of responsibility towards nature and sustainable practices from a young age is crucial for protecting the planet. Their focus on simple, actionable practices suggests an understanding of toddlers’ developmental capabilities. By embedding sustainability in daily interactions, they aim to cultivate early awareness and appreciation for the environment.

#### 3.1.2. Views of Toddlers’ Capacity to Engage in EfS

The teachers expressed strong confidence in toddlers’ ability to engage with EfS concepts. T1 contended that “They can understand and learn what we talk about even if they are one year old. We can see that they inspire their sisters, brothers, moms, and dads. Even grandma and grandpa…”. T3 reinforced this viewpoint: “We must start with the smallest children. They will be future citizens. The children can learn about this differently and repeat it daily. Because we are their first teachers, we must believe in them”. T2 mentioned incorporating simple practices into daily routines to facilitate learning: “We include activities like sorting waste and composting to help them understand”.

The teachers believed that toddlers are not only capable of understanding sustainability concepts but can also influence their families. They emphasised the importance of repetition and daily practice, recognising that consistent exposure reinforces learning. Their approach aligns with pedagogical theories that advocate for early education to shape lifelong attitudes and behaviours.

The findings indicate that preschool teachers view EfS as an essential component of early-childhood education, focusing on environmental stewardship through everyday practices. They believe toddlers possess the capacity to grasp and engage with sustainability concepts and that early education plays a pivotal role in shaping future generations. By integrating EfS into daily routines, the teachers aim to foster a foundation for sustainable thinking and actions from a young age.

### 3.2. Teachers’ Daily Practices

#### 3.2.1. The Social and Cultural Dimension of Sustainability

The social and cultural dimensions of sustainability were prominently represented through books, pictures, toys, and the promotion of social-cultural diversity, equality, and equity.

##### Representations in Books, Pictures, Toys, Etc.

In the toddler group, books, images, and materials were intentionally selected to avoid cultural and social stereotypes, for example, picture books portraying women in non-traditional gender roles, such as scientists, engineers, and astronauts, challenging the stereotype that certain jobs are only suitable for men or women. Similarly, toy sets included a diverse range of characters with different skin colours, disabilities, and backgrounds, reflecting the diversity of the world around them. The children were frequently exposed to these materials, organised for easy access in the classroom, toilet, hallway, and garden.

The teachers often read the aforementioned books to the children, who not only showed interest but also actively engaged in exploring them independently. During observation on 10 February 2024, one of the children was particularly drawn to a new book about disabled children. T2 invited the child to investigate the book with them and read it aloud. In the interviews, T2 mentioned

*“In the toddler group, we consciously choose books, images, and materials that challenge cultural and social stereotypes. We aim to foster an inclusive and respectful environment where children can learn about different cultures, traditions, and lifestyles from a young age”.*

The teachers’ daily practices demonstrate a deliberate effort to incorporate sustainability’s social and cultural dimensions into the learning environment. By selecting materials that challenge traditional stereotypes and represent diverse cultures and abilities, the teachers promoted values of inclusion and respect. The presence of books featuring women in STEM roles and toys depicting characters with various backgrounds allowed the children to see a broader spectrum of societal roles and identities.

The observation of T2 engaging with a child interested in a book about disabled children illustrates how teachers facilitate discussions on diversity and inclusion. By actively participating in the child’s exploration, the teacher reinforces the importance of understanding and accepting differences. Table 3 presents examples of materials and activities used to promote social and cultural diversity.

##### Social and Cultural Diversity

Local documents and plans emphasise the importance of cultural diversity. As stated in Lpfö 18,

*“The preschool is a social and cultural meeting place that should promote children’s understanding of the value of diversity. Awareness of different living conditions and cultures can help to develop an ability to understand and empathise with other people’s conditions and values”.*

[33] (p. 6)

The curriculum highlights that children’s understanding of languages and cultures should be enhanced. In observations, it was noted that multilingual children were encouraged to use their mother tongues, and the teachers acknowledged that the children spoke multiple languages. For instance, during circle time, they counted numbers first in Swedish, then in Arabic, and finally in Turkish, acknowledging the presence of a researcher from Turkey. This practice emphasised the value of multilingualism in the classroom and helped the children feel confident that their mother tongues held value.

**Figure 1:**
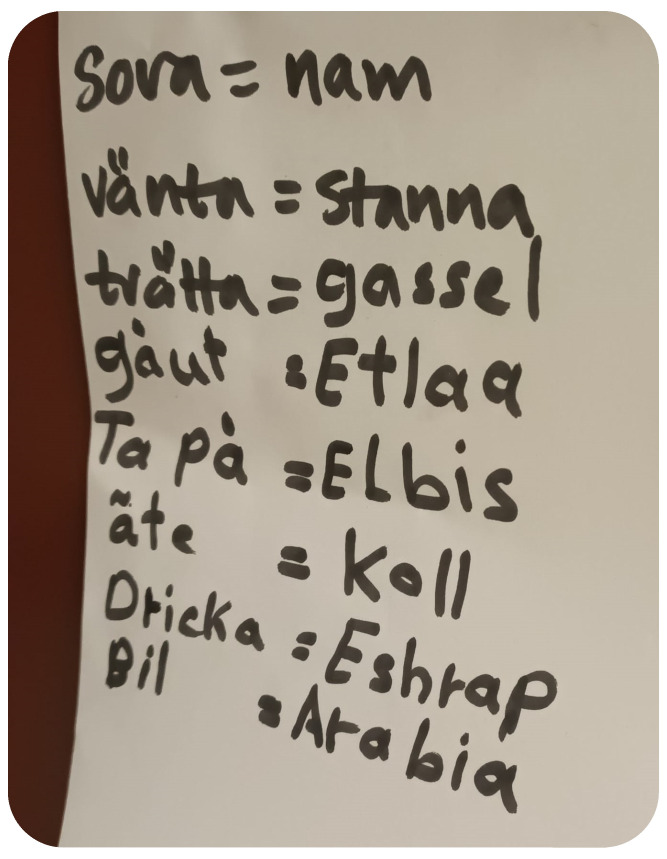
A note left by preschool teachers to remind the children of their mother tongues. In the figure, several Swedish words are paired with their Arabic equivalents to demonstrate bilingual vocabulary. For example, *sova* (to sleep) is translated as *nam*, while *vänta* (to wait) is matched with *stanna*. Similarly, *tvätta* (to wash) is paired with *sassel*, and *gå ut* (to go out) is translated as *etlaa*. The Swedish phrase *ta på* (to put on) corresponds to *elbis*, and *äta* (to eat) is rendered as *koll*. Additionally, *dricka* (to drink) is translated as *sshrap*, and *bil* (car) is shown as *arabia*. These translations illustrate a direct linguistic connection between the two languages, highlighting basic everyday vocabulary.

The teachers endeavoured to learn essential words in the children’s mother tongues to facilitate communication, especially when multilingual children had difficulties communicating in Swedish (Please see Figure 1). For example, during an observation on 3 December 2023, T2 attempted to communicate with a child in her mother tongue to ask if she wanted water. In conversations with teachers, T1 emphasised: “*Our responsibility is to understand and support the children, striving to provide an inclusive learning environment*”.

Awareness of different living conditions and traditions was also highlighted in observations on 28 November 2023. Additionally, there was a child in the classroom with special needs, and teachers made concerted efforts to understand and accommodate these needs, fostering an inclusive environment that respects and values individual differences. Table 4 presents the practices about social and cultural diversity.

##### Equality and Equity

In the classroom, numerous visuals related to the UN Convention on the Rights of the Child were displayed, including posters, pictures, and brochures. T2 commented on how they value children’s rights:

*“We sometimes open all the doors at our department. Children can freely visit all the rooms and play whatever they want. We organise our learning environment based on their interests and convenience. For example, we had a bird project. We built a nest and started to observe it in the classroom. We realised some children had difficulty seeing the birds entering the nest. Therefore, we decided to move all the tables near the windows. So, all can observe during breakfast or lunchtime. We also put a platform on the wall to make it visible outdoors to shorter children”.*

On 20 November 2023, we observed the Children’s Rights Day celebration. The children learned a song about their rights. In subsequent observations, it was noted that some children memorised and sang the song during playtime. We also observed children participating in decision-making processes. For example, on 22 March 2024, the children chose what to eat for lunch. Teachers hung pictures of meals on the wall. After an explanation, the children used pins to indicate their preferred meals. These meals were then scheduled to be cooked at the school’s planned time. Table 5 presents the practices about equality and equity. 

##### Overall Integration of the Social and Cultural Dimension

The preschool’s approach effectively integrates the social and cultural dimensions of sustainability into early-childhood education through intentional, inclusive, and age-appropriate practices. By promoting diversity, equality, and inclusion through carefully selected materials, multilingual activities, and participatory decision-making, the initiatives foster an environment where all children feel valued and respected. Collaboration with families and alignment with the curriculum amplify the impact, demonstrating a comprehensive strategy for nurturing social and cultural sustainability from a young age.

#### 3.2.2. The Economic Dimension of Sustainability

The economic dimension of sustainability was primarily represented in the preschool settings through practices of consumption, reuse and sorting, co-use, and the sharing and redistribution of materials and resources.

##### Consumption

In the preschool environment, various visual aids such as charts, graphs, and infographics were utilised to promote sustainable practices among the children, particularly in regard to reducing the consumption of water, paper, and food. Signs like “Take 1” on toilet paper dispensers encouraged mindful use. Conservation became integral to the children’s daily routines as they actively participated in activities to conserve materials and resources such as water, electricity, and paper. Teachers supported children in minimising food waste by encouraging them not to leave meals on their plates.

On 1 February 2024, a new project regarding food waste reduction was initiated. Teacher T1 prepared a chart to record measurements of leftover food on each child’s plate. After breakfast, one child collected all the leftover food into a cup, which the teacher then weighed. The teacher communicated the amount to the children simply, discussed the findings with them, and motivated them to reduce food waste by leaving less food on their plates.

**Figure 2:**
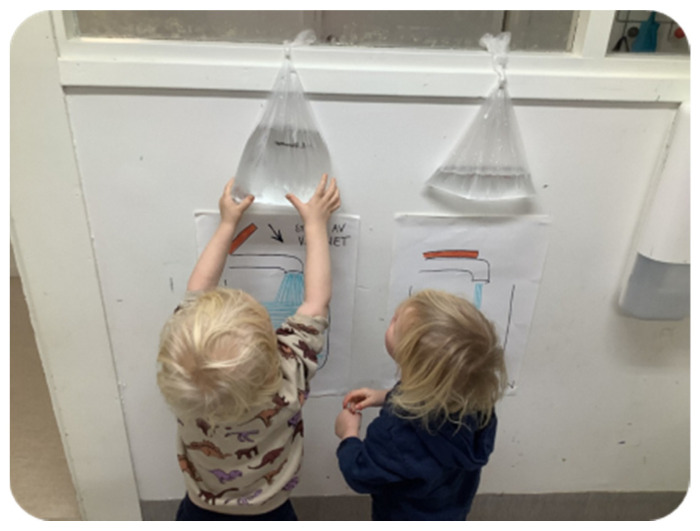
A poster about water conservation.

The teachers consistently encouraged children to use less water while washing their hands. On 7 February 2024, an experiment concerning water conservation was conducted, featuring illustrative images emphasising the importance of water. The experiment was divided into two phases. First, the children were asked to wash their hands as usual, fully opening the tap. The water used by one child was collected in a bottle to measure the quantity. Then, the children were instructed to open the tap only partially while washing their hands. The water used was again collected in a separate bottle. By comparing the two bottles, the children could visually understand the substantial amount of water wasted when the tap was fully opened. This tangible demonstration effectively highlighted the importance of careful water use. Following the experiment, the teacher displayed a poster about water conservation in the bathroom (Figure 2) as a constant reminder.

During an interview, Teacher T2 commented on their support for the “Getting Children Free from Diapers Earlier” project run by OMEP (World Organization for Early-Childhood Education):

*“It is alarming that children nowadays use diapers much longer than before, which is not good for them and not sustainable for our environment. As preschool teachers, our goal is to help children become nappy-free earlier. We collaborate with families and encourage children by introducing a potty early on in our group, creating an awareness about sustainability”.*

By promoting earlier potty training, the preschool aims to reduce both environmental impacts and economic costs associated with use of diapers, which pose a sustainability challenge due to their cost and the significant waste they generate. Please refer to Table 6 for a summary of the findings on practices aimed at reducing consumption.

##### Reuse and Sorting

The toddlers were actively encouraged to participate in various activities related to reusing and sorting waste, making it an integral part of their daily routine at preschool. For instance, after every meal, they disposed of their food scraps in the compost bin—a customary mealtime practice. Both the classroom and the school garden featured several recycling bins. The children knew which type of waste belonged in each bin and used them regularly. Every day, a group of children and a teacher would dispose of compost and other waste in designated containers in the garden. On Fridays, they discarded paper and other recyclable items. Additionally, the preschool had a storage area where they collected reusable materials like egg cartons and milk boxes for future use. The children used reusable containers and bottles for art activities, and books about reusing and sorting were made available to educate them further.

The school partnered with a company to spark children’s interest in reusing and sorting. This company created animated characters called “Sopsamlarmonster” (garbage-sorting monsters). Each monster had unique movements or exercises reflecting its characteristics. They resided in the forest near recycling centres and were responsible for collecting specific types of trash. There were 19 Sopsamlarmonsters, each specialising in a particular kind of waste; for example, “Metallika” collects metal, “Pappis” gathers old paper packaging, “Kompostina” collects food scraps, and “Flamman” takes items that no one else wants. The teachers incorporated these monsters into storytelling, songs, crafts, and role-playing activities. These characters entertained the children while educating them about reusing and sorting, promoting physical activity and interactive play.

During the interviews, T3 emphasised that using these monster characters has been effective in motivating young children to sort their garbage:

*“The children have demonstrated the ability to learn which items belong in which bins and have clearly understood how to differentiate between them. Incorporating characters, stories, songs, and role-playing in Education for Sustainability is an effective way to make the concept more tangible and engaging for younger children”.*

The children also exhibited adaptive behaviours related to reusing and sorting. On 1 February 2024, we observed a child who dropped a piece of apple while eating. Without guidance from a teacher, the child picked up the apple piece from the ground and placed it in the compost bin. On 22 November 2023, one of the teachers brought an old cabinet to the classroom. T3 explained to the children that she used this cabinet in her kitchen but no longer needed it after moving to a new house. She thought it would be a good idea to bring it to the classroom so the children could use it as a sitting bench, a car, or anything else they imagined. The children were delighted and even played hide-and-seek around it. They began using it as a sitting bench and climbing area in the classroom. There was also a rainwater storage system in the garden. On 4 March 2024, a teacher and a group of children went outside to collect water from the rainwater storage and used it to water the plants in the classroom. We noticed that the children and teachers actively used the stored water for various purposes. Table 7 presents a summary of reuse and sorting practices.

##### Co-Use, Sharing, and Redistribution of Materials and Resources

Toddlers at the preschool were encouraged to share and redistribute materials and resources, making it a fundamental part of their school experience. A large cabinet at the entrance served as a communal space where teachers, children, and parents actively contributed clothing. Teachers hung clothes left at school from previous years, while parents added clothes and shoes that had become too small for their children. Children in need and teachers from other units could freely take items from this cabinet. For example, on 16 February 2024, a girl found her shoes were wet and could select another pair from the cabinet. Similarly, on 21 March 2024, when a boy did not have enough clothes to change into, T3 helped him choose attire from the collection. This practice ensured that all children had access to necessary clothing while also promoting the reuse of resources.

In addition to swapping clothes, teachers organised visits to the library with the children, where they exchanged books (12 April 2024). T3 stated the following:

*“This practice is an excellent way to promote reading and sharing educational resources. It is a great example for our toddlers to understand the meaning of sharing. When we all share, we always have more”.*

These library visits encouraged literacy and taught children the value of sharing and collective use of resources.

T1 shared her personal experiences with the sharing economy. Growing up on a farm, she recalled sharing materials and resources: “*We used to give meat to our neighbour, and I’d get some milk back. I believe this bartering system allowed everyone access to different resources and ensured that nothing went to waste*”.

She added that she still practices this sharing approach by swapping clothes with friends and family instead of buying new ones, and she strives to inspire families and children to do the same. Please refer to Table 8 for a summary of the findings on co-use and sharing practices.

##### Overall Integration of the Economic Dimension

The preschool’s approach effectively integrates the economic dimension of sustainability into early-childhood education through practical, engaging, and age-appropriate methods. The initiatives educate children and produce tangible economic and environmental benefits by focusing on consumption reduction, waste minimisation, and resource conservation. Collaboration with families and participation in larger projects amplify the impact, demonstrating a comprehensive strategy for fostering sustainability from a young age.

#### 3.2.3. The Ecological/Environmental Dimension of Sustainability

The ecological and environmental dimension of sustainability was prominently represented through the subtitles about nature, protecting and caring for the environment, and integrating nature into the preschool experience.

##### About Nature

The classroom offered a variety of materials and resources focused on nature and the environment. Numerous books, cards, toys, and other educational materials related to nature were made available for the children to explore. The classroom was adorned with pinecones, dried leaves, flowers, and branches, bringing elements of the outdoors inside. Bug observation kits and magnifying glasses were also provided, encouraging the children to engage actively with natural specimens.

Teachers consistently organised the classroom to foster a stronger connection between the children and nature. For example, on 11 March 2024, T1 and T2 rearranged the tables and chairs to give all the children an unobstructed view of the outdoors. This allowed them to observe a bird’s nest and the birds living there, further strengthening their connection with the natural world. We observed a variety of projects and activities related to water, weather, planting, and birds, in which the children were encouraged to participate regularly. One inspiring initiative was a plantation project designed to introduce the children to scientific concepts about nature.

On 21 March 2024, the children were thrilled to participate in a planting activity during circle time. The teachers used a puppet to tell a story about a growing flower, capturing the children’s attention and sparking their curiosity. They then conducted a long-term science experiment involving planting seeds and observing the plants’ growth over time. Throughout the process, the teachers guided the children on properly watering the plants and placing them in spots with adequate sunlight. Over the following days, the children eagerly observed the plants’ changes and continued caring for them. By the beginning of summer on 3 June 2024, they began to enjoy the produce they had grown.

As part of their curriculum, the school also had a bird project designed to teach children about different bird species and their behaviours. An actual nest on a tree behind the classroom allowed the children to observe birds coming and feeding. During an interview, T2 provided the following explanation:

*“During the project, the children listened to various bird voices and learned to identify different species based on their unique sounds. We also involved them in role-playing activities about birds, where they wore different masks and hats to represent different species. This helped them understand the characteristics and behaviours of different birds. To make the learning experience more interactive, we created a big bird nest from cardboard for the children to enter and role-play as birds. The classroom was also arranged to optimise the view of the birds and their nests. The children could observe how birds build their nests and care for their young. Overall, the bird project was an excellent way for the children to learn about nature and the importance of preserving different species. I believe the children developed an appreciation for birds and their unique characteristics, which will stay with them for years”.*

Table 9 present a summary for the practices about nature.

##### Protecting and Caring for Nature

In our observations, we noted that teachers actively aimed to raise children’s awareness about protecting and caring for nature. On a rainy day (22 March 2024), a large piece of cardboard was found in the school garden. T2 invited interested children to discuss what would happen if the cardboard remained in the garden and what they should do with it. Together, they brought it into the classroom to let it dry. After it dried a few days later, they placed it in the recycling bin. In an interview, T1 shared an example:

*“Today, the children found a worm and said, ‘Oh, look! It’s tiny. We need to take care of it. We can put it in our planting area because it can live there.’ And they carefully moved the worm. They know there’s air and food for it in the planting area. We don’t just do activities about this; we also talk about it during their play, mealtime, whenever we find an opportunity. Then, a child found a snail while playing and said, ‘We have to put the snail here,’ he did. We started singing the song ‘Little snail, watch out, watch out’ They understand that they are big, but snails are small, so we need to take care of them and protect them”.*

T1 added the following:

*“Taking care of nature is crucial. We should pay attention to the smallest creatures, such as a lonely spider, and interact with them respectfully. It’s important to recycle and not litter. We must be careful not to harm tiny creatures like ants and avoid stepping on them. When we encounter animals like spiders or ants in our room, we should handle them carefully and release them outdoors unharmed. We must take care of animals since they’re essential to nature. Animals in their natural habitat should be welcomed, not feared. It’s crucial to understand the significance of nature and animals and treat them with care and respect”.*

The toddlers were also encouraged to identify environmental problems in nature, and the teachers motivated them to protect and care for their local surroundings. During a forest visit on 2 April 2024, the children wanted to pick up trash they found on the ground. They returned it to the classroom and placed it in the appropriate recycling bins. Similarly, on 10 April 2024, the children saw a piece of paper while playing in the school garden. They picked it up, told the teacher that it did not belong in nature, and put it in the recycling bin. T3 mentioned that the children’s actions inspire their families:

*“When the children start picking up trash from the ground, their parents also become interested in doing the same. One parent shared an incident with me about their forest walk where their child spotted water bottles on the ground and suggested that they collect them and give them to the teacher for recycling”.*

Table 10 represents the practices about nature protection and care for nature.

##### Preschool and Nature

**Figure 3:**
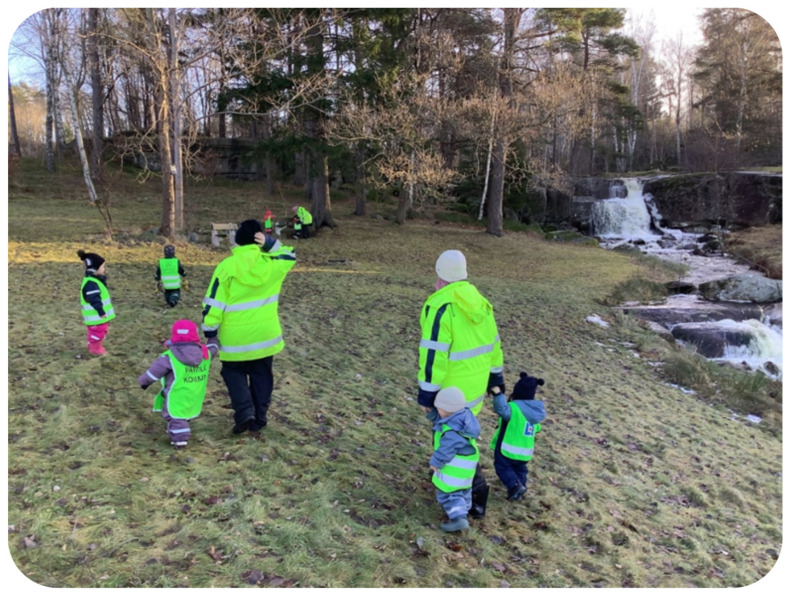
The children are visiting the forest.

The toddlers were given numerous opportunities to spend time in and explore nearby natural parks, forests, and local environments throughout different seasons of the year (Figure 3). They regularly engaged in activities that allowed them to discover the natural world firsthand. T1 made the following remark:

*“Even though they are very young, we always dare to walk in the forest. It can be challenging to move around with such young children, but we see how happy they are in the forest, how much they enjoy smelling the flowers, how they wonder where the flowers are in the winter, and how surprised they are when the flowers bloom in the spring”.*

The children spent most of their time outdoors. The school is near a forest and a small waterfall, which the children often visit. On 30 November 2023, they visited the waterfall and listened to the soothing sound of the water. The children were fascinated by the experience, and the teachers took the opportunity to discuss the different animals living in and around the water. The group even talked about various fish species found in the ocean.

During an interview, T2 shared insights from a forest trip where a child noticed that the swamps disappear when spring arrives and flowers bloom. She provided the following account:

*“Recently, we also went to the same area to gather flowers. We visited the same place during winter when Lion shouted, -What happened to the swamps? They’re gone, and the flowers are here.-. We are always mindful of how nature changes based on the weather and observe and discuss it with each other. For instance, we might say, -See that tree? It’s brown now, but it’ll be greener in the spring. Can you smell the flowers?- We always try to be observant and appreciate the beauty of nature around us”.*

Please refer to Table 11 for a summary of the results on preschool and nature.

##### Overall Integration of the Ecological/Environmental Dimension

The preschool integrates sustainability’s ecological and environmental dimensions into early-childhood education through immersive, hands-on, and age-appropriate activities. By bringing nature into the classroom, making the children engage in outdoor exploration, and fostering a culture of care and respect for the environment, the initiatives educate children and instil values of environmental stewardship. Collaboration with families and the community amplifies the impact, demonstrating a comprehensive strategy for fostering ecological sustainability from a young age. Please see Table 12 for the key findings.

## 4. Discussion

This discussion was constructed around three key ideas from the findings: “Toddlers learn habits that are important for sustainability, experiences are the foundation for sustainability knowledge in early years, and teachers’ role is central—in what ways?”.

### 4.1. Toddlers Learn Habits That Are Important for Sustainability

The findings from this study illustrate how early-childhood education settings can foster habits essential for promoting sustainability concepts among toddlers. The study revealed that toddlers could participate in daily sustainability practices, highlighting the importance of early exposure in fostering lifelong sustainable behaviours and the significant impact of early habits.

Habits are fundamental in shaping our daily actions and can pose significant challenges when promoting sustainability [45]. Chauhan et al. (2012) emphasised integrating sustainable choices into daily routines and modifying habits to align with sustainability practices [46]. By incorporating simple, everyday actions—such as saving water, recycling, and picking up garbage—sustainability becomes accessible and achievable for toddlers. These practices help toddlers develop habits foundational to sustainable living.

It is essential to recognise that our unsustainable behaviours are shaped not only by current decisions but also by past choices that have solidified into strong habits and lifestyles [45]. Therefore, developing habits that promote a sustainable life from toddlerhood provides an ideal foundation for maintaining sustainable practices throughout life [10].

Sustainability issues should be embedded in preschools’ daily routines [47]. For instance, activities like measuring food waste and encouraging conservative water use during handwashing teach children about resource conservation in tangible ways. These early experiences provide the basis for knowledge, shaping toddlers’ understanding and behaviour regarding the importance of sustainability. Encouraging children to use less water and minimise food waste aligns with the principles of sustainable consumption habits highlighted by the United Nations Sustainable Development Goals [48].

Davis (2005) reported that very young children developed water conservation habits and transferred these habits to their homes. Similarly, in the current study, sustainability practices like picking up litter in the forest often influenced families to adopt similar behaviours [49]. As Buil et al. (2019) pointed out, different stakeholders—such as children, parents, teachers, managers, and associations—can collaborate to enhance education on sustainable development in early childhood, benefiting all by embedding habits essential for sustainability [47]. These activities help children develop habits that promote a sustainable world and provide them with a variety of hands-on learning experiences [45], which are effective in building understanding and retention [50].

### 4.2. Experiences as the Foundation for Sustainability Knowledge in Early Years

This study provided numerous examples where hands-on activities enhanced toddlers’ interest in sustainability issues. Projects such as planting seeds, observing plant growth, and participating in bird-watching activities helped the toddlers connect with nature and understand ecological processes. These experiences fostered curiosity and instilled a sense of responsibility and care for the environment. For instance, the bird project, where children observed actual bird nests and identified bird species by their calls, enriched their knowledge and appreciation of biodiversity. Similarly, waste-sorting and composting activities taught the toddlers practical skills and reinforced the importance of waste management.

Including books, pictures, and toys depicting diverse characters and challenging stereotypes is instrumental in fostering an inclusive mindset. Regular exposure to such materials helps toddlers understand cultural and social differences [51]. Allowing toddlers to use their mother tongues and incorporating different cultural practices into the curriculum enhances their understanding of diversity [52]. Teachers’ efforts to learn essential words in various languages and acknowledge the linguistic diversity in the classroom fostered an inclusive environment. These experiences help children develop empathy and appreciation for different cultures, laying a foundation for social understanding [53].

Promoting equality and equity through visuals about the UN Convention on the Rights of the Child and involving children in decision-making empower them and teach them about their rights [54]. Activities like choosing meals and participating in classroom decisions help children understand social justice and the importance of inclusivity. These experiences instil values of fairness and respect from an early age.

Experiences serve as the foundation for sustainability knowledge in the early years, crucially fostering curiosity, environmental stewardship, social understanding, and a sense of responsibility among toddlers. This establishes a solid basis for lifelong learning and empathy. Building on the premise that experiences form the bedrock of early knowledge, it is evident that teachers’ roles are central in facilitating these experiences, guiding children’s learning, and modelling inclusive and sustainable practices.

### 4.3. The Teacher’s Role Is Central—In What Ways?

The role of teachers in fostering sustainability among toddlers is crucial [16]. As role models, they demonstrate sustainable behaviours early on, such as conserving water, picking up litter, and caring for animals. In the current study, teachers provided toddlers with concrete examples of sustainable living through their actions and daily routines. They promoted sustainable consumption and resource management by implementing food waste reduction projects and conducting water conservation experiments.

According to Davis and Elliot (2023), teachers embed sustainability in early-childhood education by involving children in practical activities that encourage understanding and action regarding environmental issues [8]. Teachers facilitate reusing materials and sorting waste. Bandura’s (1986) [55] concept of observational learning highlights that children acquire new behaviours by observing others, making the teacher’s role essential in promoting sustainability.

In this research, teachers’ efforts extended beyond the classroom, inspiring toddlers to influence their families and communities. The children often shared what they learned about sustainability with their parents, leading to broader community engagement in sustainable practices. This ripple effect is crucial for building a culture of sustainability, as emphasised by Vandenbroeck, Roets, and Snoeck (2009), who highlight the role of early-childhood education in shaping societal values and practices [56].

The findings also revealed that teachers actively challenged cultural and social stereotypes by selecting books, images, and materials that promote inclusivity and an understanding of different cultures and traditions. According to Siraj-Blatchford et al. (2010), preschool teachers play a vital role in developing inclusive settings that reflect diversity and promote social equity [57]. The teachers in this study supported multilingualism, cultural awareness, and children’s rights, incorporating principles from the UN Convention on the Rights of the Child. This approach aligns with Cummins’ (2001) findings on maintaining and valuing children’s first languages as a foundation for learning additional languages and fostering a sense of cultural identity [58].

Teachers are central to promoting sustainability in toddlerhood. They lay the foundation for a sustainable future through inclusive practices, cultural awareness, equity, resource management, connection with nature, modelling behaviours, and community engagement. There is an extensive amount of research, e.g., Refs. [30,31,59], supporting these practices, underscoring the critical impact of teachers in shaping young children’s understanding and commitment to sustainability.

## 5. Limitations and Future Directions

While this study provides valuable insights into the role of early-childhood education for sustainability (EfS) within Swedish preschool settings, several limitations must be acknowledged. This research is based on a case study with a limited sample size and a specific context, so it may not represent broader educational settings. Additionally, the study relies on self-reported data from teachers, introducing potential bias, as their descriptions may reflect aspirations rather than actual classroom activities. The findings do not track the long-term impact of EfS on toddlers’ behaviours and attitudes, and differences in resources, administrative support, and cultural attitudes across educational settings may influence the effectiveness of EfS practices.

Further research is needed to address these limitations and build on the current findings. Longitudinal studies assessing the lasting influence of early EfS initiatives on children’s behaviours, attitudes, and knowledge would provide more robust insights. Comparative studies across diverse regions, cultures, and educational contexts could identify best practices that are adaptable to various settings. Exploring the collaborative roles of families and communities in supporting sustainable practices initiated in preschool may also offer valuable perspectives on creating cohesive and supportive environments for sustainability education. Incorporating quantitative measures alongside qualitative data would enable a more comprehensive evaluation of EfS effectiveness, including metrics such as pre- and post-assessments of children’s sustainability knowledge and behaviour changes. While this study underscores the potential contribution of early EfS initiatives to global sustainability goals, extensive research is necessary to generalize these findings and determine their applicability across diverse educational frameworks.

## 6. Conclusions

This study highlights the critical role of early-childhood education settings in fostering sustainable habits among toddlers. By integrating simple, actionable sustainability practices—such as saving water, recycling, and caring for living creatures—into daily routines, preschool teachers can instil foundational habits that promote lifelong sustainable behaviours. The findings underscore that habits formed during toddlerhood are pivotal, as they can either contribute to or mitigate the challenges of promoting sustainability later in life.

Experiential learning emerged as a fundamental component in embedding sustainability into early-childhood education from toddlerhood. Hands-on activities like planting seeds, observing plant growth, participating in bird-watching projects, and engaging in waste sorting not only enhanced children’s curiosity but also fostered a deep sense of responsibility and care for the environment. These experiences facilitated an understanding of ecological processes and the importance of biodiversity, laying a strong foundation for environmental stewardship from an early age.

Moreover, this study revealed that teachers who supports children’s understanding, play a central role in this developmental process. By serving as role models and actively demonstrating sustainable behaviours, teachers influence not only the children they teach but also their families and the broader community. Their efforts in challenging cultural and social stereotypes, promoting inclusivity, and supporting multilingualism contribute to a comprehensive approach to sustainability that encompasses environmental, social, and cultural dimensions.

In conclusion, fostering sustainable habits and knowledge among toddlers is not only feasible but essential. Early-childhood educators, through intentional practices and experiential learning opportunities, can cultivate a generation that is more conscious of and committed to sustainability. By prioritizing EfS from the earliest years, we lay the groundwork for more sustainable futures.

## Figures and Tables

**Table 1:** Characteristics of the participants.

Participants	Gender	Age	The Educational Level	Years of Teaching
T1	F	47	University	16
T2	F	53	University	13
T3	F	53	Collage	3.5

**Table 2:** Data collection and analysis process.

Research Questions	Data Collection Techniques	Data Analyses
What are the views of preschool teachers on ECEfS in a toddler group at a Swedish preschool?	Interviews Document analysis	Thematic analysis following Braun and Clarke’s (2006) six-phase process [36].
		Employing open coding to identify expressions relevant to recurring patterns or themes.
		Grouping codes into potential themes and collating relevant data.
		Using MAXQDA software for word frequency checks to refine codes.
		Aligning codes with the relevant literature.
What are the daily practices employed to foster sustainability among toddlers at a Swedish preschool?	Interviews Observation Document analysis	Thematic analysis following Braun and Clarke’s (2006) six-phase process [36].
		Employing open coding to identify expressions relevant to recurring patterns or themes.
		Grouping codes into potential themes and collating relevant data.
		Using MAXQDA software for word frequency checks to refine codes.
		Aligning codes with the OMEP ESD Rating Scale (ERS-SDEC).

**Table 3:** Materials and activities promoting social and cultural diversity.

Materials/Activities	Description	Observed Impact
Picture books featuring women in STEM roles	Books portraying women as scientists, engineers, and astronauts	Challenged gender stereotypes; engaged children’s interest
Diverse toy sets	Toys representing various skin colours, disabilities, and backgrounds	Promoted inclusion; allowed children to identify with diversity
Multilingual books and labels	Books and labels in different languages present in the classroom	Encouraged use of mother tongue; valued linguistic diversity

**Table 4:** Practices about social and cultural diversity.

Practice	Description	Observed Impact
Multilingual activities	Counting numbers in different languages during circle time	Showed the value of linguistic diversity; boosted children’s confidence in using their mother tongues
Learning essential words	Teachers learning words in the children’s mother tongues	Improved communication; strengthened teacher–child relationships
Inclusive support for children with special needs	Accommodating children with special needs	Fostered an inclusive environment; helped promote respect for individual differences
Cultural awareness activities	Highlighting different living conditions and traditions	Enhanced empathy; broadened children’s understanding of diverse cultures

**Table 5:** Practices promoting equality and equity.

Practice	Description	Observed Impact
Visual displays of children’s rights	Posters and materials related to the UN Convention on the Rights of the Child	Raised awareness of rights; fostered discussions
Adaptive learning environment	Adjusting classroom setup to meet children’s needs (e.g., bird project)	Ensured equal access; addressed individual needs
Participation in decision-making process	Children choosing meals and activities	Empowered children; promoted autonomy and responsibility
Celebrating children’s rights day	Teaching the toddlers songs and activities about children’s rights	Reinforced understanding of rights; engaged children in meaningful learning

**Table 6:** Practices about consumption.

Practice	Description	Observed Impact
Water conservation experiment	Comparing water usage between fully opened and partially opened taps	Children became more mindful of water usage and gained visual understanding of conservation
Food waste measurement	Weighing leftover food and discussing results with children	Reduction in food waste: children were more conscious about taking appropriate portions
Diaper use reduction initiative	Encouraging earlier potty training in collaboration with families	Decreased diaper usage; cost savings; environmental benefits

**Table 7:** Reuse and sorting practices.

Practice	Description	Observed Impact
Waste-sorting activities	Asking children to sort recyclables into designated bins	Increased understanding of recycling; active participation in waste management
Composting	Disposing of food scraps in compost bins after meals	Awareness of organic waste recycling; contribution to garden compost
Sopsamlarmonster integration	Using animated characters to teach about recycling	Enhanced engagement in learning about sorting; improved retention of recycling concepts
Reuse of materials	Collecting reusable items for art activities; repurposing old furniture	Promotion of creativity; enhanced understanding of reusing materials instead of discarding
Rainwater storage usage	Collecting rainwater for watering plants	Comprehension of water conservation; practical application of resource reuse

**Table 8:** Co-use and sharing practices.

Practice	Description	Observed Impact
Clothing exchange cabinet	Communal cabinet where clothing and shoes are shared among children, teachers, and parents	Ensured all children had necessary clothing; promoted reuse and sharing values
Book exchange at library	Regular visits to the library to exchange books	Encouraged literacy; taught the value of sharing educational resources
Sharing-economy examples	Teachers sharing personal experiences of bartering and swapping resources	Inspired families to adopt sharing practices; reinforced community values

**Table 9:** Practices about nature.

Practice	Description	Observed Impact
Classroom nature materials	Inclusion of natural items (pinecones, leaves) in the classroom	Enhanced connection with nature; sensory engagement
Planting project	Planting seeds and observing growth over time	Understanding of plant life cycles; responsibility in caring for plants
Bird project	Observing actual bird nests and role-playing as birds	Awareness of bird species; appreciation for wildlife
Rearranging classroom layout	Adjusting the classroom to provide unobstructed outdoor views	Increased observation of nature; integration of indoor and outdoor learning

**Table 10:** Practices about nature protection of and care for nature.

Practice	Description	Observed Impact
Cardboard-recycling initiative	The children found and decided to recycle a piece of cardboard	Understanding of recycling; problem-solving skills
Caring for small creatures	Respectful interactions with worms, snails, spiders, ants	Development of empathy; appreciation for all living things
Trash collection during forest visits	Children collected trash found in nature and recycled it	Environmental responsibility: proactive care for the environment
Family influence	Children’s actions inspiring sustainable practices at home	Extended impact beyond preschool; family engagement in sustainability

**Table 11:** Preschool and nature.

Practice	Description	Observed Impact
Forest walks	Regular visits to the forest and natural areas	Joy and happiness in nature; development of observational skills
Waterfall visits	Listening to water sounds; discussing aquatic life	Fascination with natural phenomena; learning about ecosystems
Observing seasonal changes	Noticing differences in nature across seasons	Understanding of environmental cycles; enhanced appreciation
Flower gathering	Collecting flowers; sensory exploration	Sensory engagement: fostering curiosity and wonder

**Table 12:** Key findings.

Main Theme	Subtheme	Key Findings
Preschool Teachers’ Views	Views of EfS in Toddler Groups	Views on ECEfS as an environmental and ecological dimension
	Views of Toddlers’ Capacity to Engage in EfS	Confidence in toddlers’ abilities to grasp ECEfS
		Importance of starting with the youngest for long-term sustainable behaviours
Preschool Teachers’ Daily Practices—Social and Cultural Dimension of Sustainability	Representations in Books, Pictures, and Toys	Inclusion of diverse materials
		Promoting diversity and inclusion
	Social and Cultural Diversity	Emphasis on cultural diversity
		Support for multilingualism
	Equality and Equity	Adaptive learning environment
		Child participation in decision-making
Preschool Teachers’ Daily Practices—Economic Dimension of Sustainability	Consumption	Active engagement in con-versation projects and mindful use of resources in daily life
		Support for early potty training
	Reuse and Sorting	Integration of reuse, sorting, and composting activities in daily life
		Collaboration with a third-party educational initiative provides a benefit
	Co-use, Sharing, and Redistribution	Encouragement of sharing and redistribution of re-sources like clothes, books, and toys.
Preschool Teachers’ Daily Practices—The Ecological/Environmental Dimension of Sustainability	About Nature	Hands-on nature projects like planting seeds and observing bird nests
		Incorporation of natural elements (pinecones, leaves) and educational materials about nature into the classroom
	Protecting and Caring for Nature	Encouraging children to recognize the impact of human actions on nature
		Extending the impact beyond the preschool by influencing families with actions like picking up trash
		Fostering empathy towards nature by caring for small creatures
	Preschool and Nature	Observation of natural changes during regular vis-its
		Fostering a connection with nature

## Data Availability

The data presented in this study are not available due to privacy and ethical restrictions.
